# Tube-Super Dielectric Materials: Electrostatic Capacitors with Energy Density Greater than 200 J·cm^−3^

**DOI:** 10.3390/ma8095301

**Published:** 2015-09-17

**Authors:** Francisco Javier Quintero Cortes, Jonathan Phillips

**Affiliations:** 1 Universidad Nacional de Colombia, Bogotá 111321, Colombia; E-Mail: fjquinteroc@unal.edu.co; 2 Physics Department, Naval Postgraduate School, Monterey, CA 93943, USA

**Keywords:** energy storage, capacitor, dielectric materials, titania nanotube arrays

## Abstract

The construction and performance of a second generation of super dielectric material based electrostatic capacitors (EC), with energy density greater than 200 J·cm^−3^, which rival the best reported energy density of electric double layer capacitors (EDLC), also known as supercapacitors, are reported. The first generation super dielectric materials (SDM) are multi-material mixtures with dielectric constants greater than 1.0 × 10^5^, composed of a porous, electrically insulating powder filled with a polarizable, ion-containing liquid. Second-generation SDMs (TSDM), introduced here, are anodic titania nanotube arrays filled with concentrated aqueous salt solutions. Capacitors using TiO_2_ based TSDM were found to have dielectric constants at ~0 Hz greater than 10^7^ in all cases, a maximum operating voltage of greater than 2 volts and remarkable energy density that surpasses the highest previously reported for EC capacitors by approximately one order of magnitude. A simple model based on the classic ponderable media model was shown to be largely consistent with data from nine EC type capacitors employing TSDM.

## 1. Introduction

At present there is a world-wide focus on improving the energy density of capacitors for a variety of reasons, from buffering power surges in order to improve battery life in vehicles and satellites to storing energy from episodic energy sources such as solar and wind. Most of the effort is focused on improving the electrodes in electric double layer capacitors (EDLC), particularly graphene electrodes. In this report we demonstrate a new type of energy storage capacitor: tube super dielectric materials (TSDM) in electrostatic capacitors (EC) enabling energy density greater than 200 J·cm^−3^.

Super dielectric materials (SDM) are defined as materials with intrinsic dielectric constants greater than 1.0 × 10^5^ at low frequency. Only two prior reports demonstrate the existence of material in this category. In both cases the material studied consisted of high surface area porous alumina powder, filled to the point of incipient wetness with water containing high concentrations of dissolved ions: boric acid in one case [1], and dissolved sodium chloride in the other [2]. In those studies the measured dielectric constants were as high as 1.0 × 10^10^ at ~1.0 × 10^−2^ Hz, meaning the materials discovered were not only SDM, but also had dielectric constants many orders of magnitude greater than any previously reported.

A simple theory was put forward to explain the existence of super dielectric behavior for materials of the type described. In brief, any non-conductive porous solid in which the pores are filled with a liquid with a sufficient concentration of dissolved ions will have a high dielectric constant. Such a multi-material mixture is a dielectric material by definition; no conduction will take place because the porous solid is insulating the solution from the conducting electrodes. The model postulates the physical basis for the high dielectric constant is the migration of the dissolved ionic species to form giant dipoles within the liquid filling the pores of the insulating material in the presence of an applied field. The theory is a modification of the classic “ponderable media” model, which, in short, is that the dielectric value of a media is proportional to the size and concentration of dipoles that will form in the media upon the application of an electric field [3]. A simple quantitative version of this theory was developed in earlier work. It predicted that the SDM would have dielectric constants thousands of times greater than that of the best solid material, barium titanate. In fact, the measured values were closer to a million times greater.

As previously discussed, the discovery of SDM has clear technological significance. Specifically, SDM can dramatically increase the electric energy density of electrostatic (ceramic) capacitors. That is, as stored energy density in capacitors is proportional to capacitance and capacitance is proportional to the dielectric constant, the unprecedented values of the dielectric constants measured for SDM are the key to the increased energy density of SDM based electrostatic capacitors. However, in the prior reports the SDMs employed were powder, filled to incipient wetness with water containing dissolved salt. The capacitors were assembled by hand, and although the dielectric constants were remarkably high, the dielectric thicknesses were 300 μm, or more. Such thicknesses are far higher than the best commercial dielectric based capacitors, *ca*. 1 μm, and not consistent with high energy density, as energy density for powder based SDM capacitors is inversely proportional to dielectric thickness squared. Clearly, the thinner the dielectric layer, the higher the energy density. Thus, to make full use of the advantages of the high dielectric constants in SDM type materials, very thin dielectric layers should be employed.

The present paper introduces a technology that enables high energy density SDM based capacitors. The earlier SDM employed wetted powders. The new version employs tube SDM (TSDM), a dense set of hollow, oriented, insulating, ceramic, micron-scale tubes filled with liquids containing a high density of ions. The fundamental prediction of the SDM theory proved to be correct. To wit: TSDM structures composed of TiO_2_ created on titanium metal using anodization, then filled with an aqueous saturated salt solution of NaNO_3_, consistently yielded energy density greater than 200 J·cm^−3^, a value orders of magnitude greater than ever observed for an electrostatic capacitor.

TSDMs, as explained in more detail later (Discussion), are fundamentally different from all other types of capacitors. For example, although they are clearly electrostatic capacitors, the measured dielectric constant is not constant, but rather increases with increasing distance between electrodes. This measured property is consistent with a simple model that arises naturally from the geometry of TSDM: the dielectric constant should increase as the square of the distance between electrodes such that tube length will not impact energy density. In contrast, the impact of salt concentration was inconsistent with predictions. The model predicts the higher the salt concentration, the higher the energy density. In practice, the relationship between salt concentration and energy density proved to be more complex.

## 2. Results and Discussion

### 2.1. Exemplary: Ten Micron Tubes with a Saturated Solution of NaNO_3_

**Figure 1:**
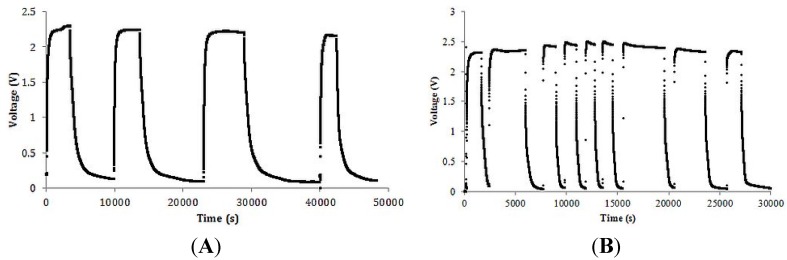
Multiple cycles. (**A**) Four full cycles of charge/discharge on the 10.6 μm, 0.925 g·mL^−1^ capacitor, 20 kΩ load (Table 1). Clearly, the maximum charging voltage the capacitor was in the order of 2.2 volts; (**B**) Nine cycles, 7 μm, 0.750 g·mL^−1^ capacitor, 20 kΩ load (Table 2). Data recorded once every second.

A typical charge/discharge curve for a capacitor made with 10.6 micron-long titania tubes and a saturated sodium nitrate solution (Table 1) is shown in Figure 1. It was found that the capacitor was capable of fully charging and discharging over many cycles with little change in the measured dielectric constant. Indeed, the dielectric constant changed by less than 15% during the four charge/discharge cycles shown (~14 h).

The individual cycles were analyzed to determine regions of constant capacitance, simply by plotting the data according to Equation (1) and determining the time and voltage regions that have a straight line.

(1) ln(VV0)=−τRC

Being *V* the voltage in the instant τ, and *V*_0_ the initial voltage during the discharge, the slope of a curve of the left side of Equation (1) *vs.* time is equal to 1/*RC*. As *R* is known and fixed, *C* is readily obtained. The value of *C*, and needed dimensional measurements, were used to determine the dielectric constant. It was generally found that there were three regions of voltage in which the capacitors displayed different, but constant, dielectric constants.

Energy density (*u*) was determined by use of the simple formula derived elsewhere [1,2]:

(2) u=0.5εε0V2t2

where ε is the dielectric constant, ε_0_ is the permittivity of free space (8.85 × 10^−12^ F·m^−1^), *V* is the maximum voltage and *t* is the thickness of the dielectric layer. The formula was applied over regions of voltage in which it was clearly shown the dielectric constant had a single value. Total energy density was computed by adding the energy density computed for each voltage region of constant dielectric constant. From the capacitance the dielectric constant is found from Equation (3).

(3) ε=tCε0A

where ε_0_ is the permittivity of free space (8.85 × 10^−12^ F·m^−1^), *t* is the dielectric thickness and *A* is the electrode surface area.

**Figure 2:**
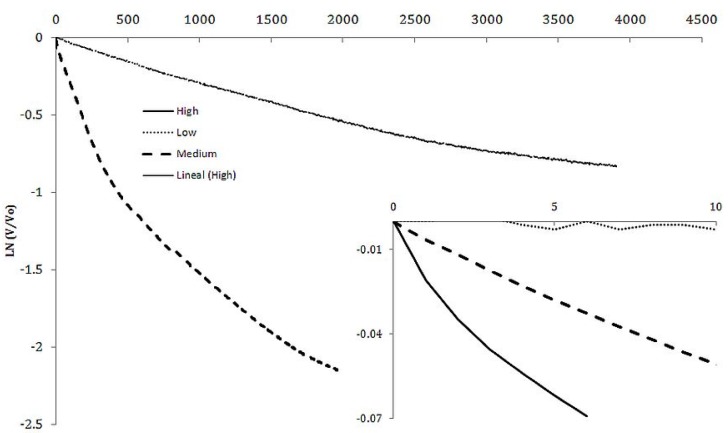
Capacitance from voltage discharge rate. The two approximately linear plots show capacitance is nearly constant over ranges of voltage. It is shown in this plot that it is greater at *Low* voltage than at *Medium* voltage. The voltages and associated capacitances are given in Table 1. Note: the shift is capacitance is sharp. There is only a short “elbow” between these constant capacitance ranges. The inset shows that in the 2.2 to 2.0 voltage region (dotted line, *High* voltage region)), the dielectric constant is about an order of magnitude smaller (Table 1).

It was found, in a fashion similar to that found in the earlier SDM studies that the dielectric constant increases as the voltage gets lower. In general there were three voltage regions of constant dielectric value, from about 2.2 volts to 2.0 volts (High), from about 2.0 volts to 0.3 volts (Medium) and from 0.3 volts to 0 volts (Low), as illustrated in Figure 2. Even for crystals in the “barium titanate family”, impedance spectroscopy shows capacitance to continuously vary as a function of voltage, sometimes, very dramatically [4,5]. Theoretically, this should be the case for all “high” dielectric constant materials [4].

The average values of capacitance and dielectric constant determined, using the *RC* time constant method, from the three discharge components of the cycles shown in Figure 2 are given in Table 1.

**Table 1:** Capacitive energy storage. Average values of dielectric constant, capacitance and energy density for the four cycles shown in Figure 1, left side. The values recorded for each individual cycle were within 15% of the average values reported. Indeed, the highest measured energy density was 280 J·cm^−3^ and the lowest 225 J·cm^−3^.

Region (V)	Dielectric Constant (±15%)	Capacitance (F)	Energy Density (J·cm^−3^)
2.2–2.0	1.81 × 10^8^	0.006	5
2.0–0.3	1.26 × 10^9^	0.045	220
0.3–0	6.60 × 10^9^	0.25	25
Total Energy Density (Entire Voltage Range)			250 ± 35

### 2.2. Impact of Tube Length and Salt Concentration

The quantitative model of TSDM predicts that energy density will not be a function of tube length, and will be a linear function of salt concentration. Nine capacitors were constructed and characterized for the purpose of testing the model (Table 2). In order to test the impact of tube length on energy density, tubes of four different lengths were tested, from 3.4 to 18 μm in length, a range of >5. In order to test the impact of salt concentration, three different salt levels were employed, the highest a saturated solution of NaNO_3_ and the lowest about two thirds of that value.

Each capacitor was put through at least four full cycles using the same protocol as described for the 10 μm tubes. The energy densities listed in Table 2 represent the average values from the discharge component of the cycles. Note the energy densities are broken down by voltage/dielectric constant regions. In some cases there is no report for energy storage above 2.0 volts because experimentally the energy storage in this range was found to be trivial. In all cases the maximum and minimum values were within 15% of the average value reported.

The most remarkable feature of the results is the consistent finding of energy densities orders of magnitude higher than any reported previously for dielectric based electrostatic capacitors. For example, the capacitors created from saturated solutions, at all tube lengths, were repeatedly found to have a net energy density above 215 J·cm^−3^, and in fact in one case ~250 J·cm^−3^.

Finally, it should be noted that control studies of behavior in the absence of salt solutions showed no measureable capacitance. That is, an anodized foil to which either (i) no salt solution had ever been added; or (ii) salt solution had been added before it was dried simply by leaving the foil in the lab for more than 10 days showed the same behavior, that of a very high resistance resistor. No capacitive behavior was observed at all. To wit: upon “charging” (*ca.* 4 volts) both virtually immediately reached the charging voltage. “Discharge” resulted in a near instantaneous drop to zero volts. For the “dried” foil (ii) above, the addition of distilled water nearly restored 100% of the capacitance. Similar behavior was observed for powder SDM [2].

**Table 2:** Summary of key capacitor parameters and data obtained from nine capacitors generated and studied.

Thickness (μm)	Salt Concentration (g·cm^−3^)	Voltage Range (V)	Dielectric Constant	Capacitance (F)	Energy Density (J·cm^−3^)
3.4	0.625	2–0.3	9.59 × 10^7^	0.009	143.53
		0.3–0	2.84 × 10^8^	0.027	9.78
		Total Energy Density (Entire Voltage Range)			153.32
	0.75	2–0.3	2.10 × 10^7^	0.002	31.43
		0.3–0	2.24 × 10^8^	0.017	7.70
		Total Energy Density (Entire Voltage Range)			39.13
	0.925	2.2–2	2.21 × 10^7^	0.002	7.11
		2–0.3	1.36 × 10^8^	0.011	203.55
		0.3–0	5.47 × 10^8^	0.043	18.84
		Total Energy Density (Entire Voltage Range)			229.50
7	0.75	2–0.3	2.06 × 10^8^	0.009	72.74
		0.3–0	1.22 × 10^9^	0.056	9.92
		Total Energy Density (Entire Voltage Range)			82.65
	0.925 *	2–0.3	5.54 × 10^8^	0.025	195.62
		0.3–0	2.44 × 10^9^	0.111	19.83
		Total Energy Density (Entire Voltage Range)			215.45
10	0.625 *	2.2–2	6.50 × 10^7^	0.002	2.42
		2–0.3	7.07 × 10^8^	0.023	122.32
		0.3–0	2.29 × 10^9^	0.073	9.12
		Total Energy Density (Entire Voltage Range)			133.86
	0.75	2–0.3	5.11 × 10^8^	0.014	88.41
		0.3–0	1.45 × 10^9^	0.038	5.77
		Total Energy Density (Entire Voltage Range)			94.19
	0.925	2.2–2	1.91 × 10^8^	0.005	7.10
		2–0.3	1.26 × 10^9^	0.033	218.00
		0.3–0	7.05 × 10^9^	0.187	28.08
		Total Energy Density (Entire Voltage Range)			253.18
18	0.925	2.2–2	2.87 × 10^8^	0.005	3.29
		2–0	4.19 × 10^9^	0.074	228.90
		Total Energy Density (Entire Voltage Range)			232.19

* These capacitors studied for internal and output resistance values.

### 2.3. Unique TSDM

Remarkably the simple equation relating capacitance to physical parameters explains virtually all research in the field. To wit:

(4) C=εε0At

It contains three physical variables, *A* area of electrodes, *t* distance between plates, and ɛ dielectric constant, that correlate to the three standard approaches to increasing capacitance. The fact that TSDM behavior is not readily explained with this equation demonstrates the uniqueness of TSDM.

Most effort is focused on increasing the electrode surface or “*A*” value. “Supercapacitors”, better known as electric double layer capacitors (EDLC), are based on increasing the electrode area. The ultimate material for this approach is likely to be graphene as it has the highest surface area per volume of any electrically conductive material. It is believed that the ultimate energy density of capacitors of this sort will be ~800 J·cm^−3^. At present, the best commercial devices have about 30 J·cm^−3^ of energy density and the best prototypes ~300 J·cm^−3^ (see Table 3). The ultimate surface area limits the ultimate energy volume.

The second standard approach is to decrease the distance between flat metal plates, that is, minimize the value of *t*, the dielectric thickness. Significant effort is underway based on this approach in order to create larger energy “capacitors on a chip” for powering micromachines, *etc.* There are now commercial capacitor “stacks” consisting of hundreds of layers, in which each capacitor in the stack has a dielectric of thickness of the order of 1 μm. Still, the energy density is less than 10 J·cm^−3^. There are three limitations: (i) the discharge voltage is a function of thickness, becoming quite low for thickness of the micron scale [5]; (ii) the dielectric material, generally barium titanate or some derivative with a practical dielectric constant no greater than 1500; and (iii) the packaging, including electrodes, is more than half the volume.

Can a capacitor be constructed to minimize t and maximize A? For example, is it possible to design a better t value into an Electric Double Layer Capacitor (EDLC)? It appears unlikely. In an EDLC or “supercapacitor”, the double layer thickness around the electrode material is a “material property” that cannot be modified. In other words, “*t*” is fixed. This is also true of the “dielectric constant” of a double layer. It is what it is. In general, it is considered [6] that carbon based electrodes have a net capacitance of ~10–20 μ/cm^2^. This is a value that clearly incorporates both thickness and dielectric constant.

Can TSDM be considered “supercapacitors”? For several reasons this is clearly not the case. First, the surface area of a titania in which half the volume is 90 nm diameter “holes” is ~20 m^2^/gm. In contrast, graphene with a surface area of ~2600 m^2^/gm is computed to have a surface area based theoretical maximum capacitance of between 500 and 800 F/gm. This alone indicates insufficient surface area for “supercapacitance”. Second, below the breakdown voltage of water the dielectric in TSDM is not conductive, but a requirement of the electrodes in EDLC is that they be very conductive.

The third standard approach is to increase the dielectric constant. Prior to our work with SDM there were no reports of dielectric constants greater than 10^4^ and most of the work was stagnant, retreading the same path: studies on barium titanate and related crystal structures. However, our efforts with powder SDM were focused on this third “standard” approach, and were extremely successful. Our work with the first-generation SDM, powder SDM, broke through the barrier of high dielectric constants. As discussed in recent publications [1,2] we have generated materials, first generation SDM, with dielectric constants as high as 1.0 × 10^10^. That is, we have demonstrated dielectric constants approximately six orders of magnitude higher than any previously measured. However, the current generation capacitors based on powder SDM are limited as energy storage devices for two reasons. First, they are a few hundred microns thick. Second, like EDLC the ultimate voltage is limited by the creation of free ions in the electrolyte outside the pores of the insulating material, which short the dielectric, due to the electrolysis of water at ~1.2 volts. This limitation is fundamental to the powder SDM.

**Figure 3:**
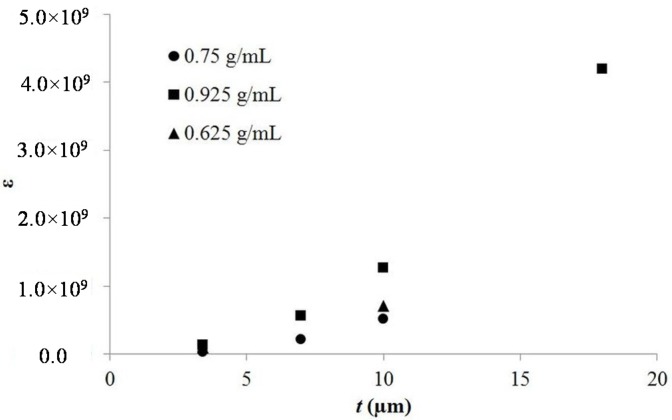
Dielectric constant as a function of thickness. This plot clearly demonstrates that unlike other materials that have a single dielectric constant, the TSDM “material” is defined by tube length. Indeed, it is clear that the dielectric constant is not a linear function of length, but proportional to length squared. Finally, note each TSDM with different tube length is effectively a unique material with a unique dielectric constant.

Second-generation SDM, or tube SDM (TSDM), consisting of anodic TiO_2_ nanotube arrays filled with concentrated aqueous salt solutions represent a fundamentally different approach to capacitance increase. This is evident because TSDM cannot be explained in full from Equation (4). Its most significant unique aspect is that all materials, including powder SDMs but not TSDMs, have a single dielectric constant. It is not a function of the structure of the material. It is an intrinsic property of the material. In contrast the dielectric constant of a TSDM increases with increasing thickness (Figure 3). It is not a simple intrinsic property of the material. Unlike a crystalline material, an anodized layer consisting of a base oxide and oxide nanotubes normal to the base oxide, there is no “symmetry” with thickness. That is, an anodized film ten microns thick is not simply a “double” of a film five microns thick. Thus, it is not necessarily the case that the measured property of a ten micron thick layer should be twice/half those of a five layer thick sample. For example, a ten micron sample is probably only slightly less light transparent than a five micron sample. In both cases only the base oxide layer, same structure in both cases, is blocking most of the light. Fill the nanotubes with salt water and this analysis is also probably true for electrical conductivity above the water breakdown voltage. That is, the bottom cap dominates the net conductivity. Heat conductivity will also not change in a linear fashion.

**Table 3:** Recent advances in high energy storage capacitors. Tube super dielectric materials (TSDM) are on par with EDLCs and far above ceramic capacitors.

Electrical Double Layer Capacitors						
Electrodes	Electrolyte	F·g^−1^	F·cm^−3^	J·g^−1^	J·cm^−3^	Reference
Activated Carbon (AC)	Aqueous	238	119 ^a^	86.4	43.2 ^a^	[7]
Graphene Aerogel	Aqueous	223	-	112	-	[8]
PbO_2_/AC	Aqueous	132	66 ^a^	180	90 ^a^	[9]
Vanadium Pentoxide	Ionic liquid	-	-	190	-	[10]
Graphene hydrogel at Nickel Foam	Aqueous	1369	-	198	-	[11]
Carbon Foam	Aqueous	-	-	227	114 ^a^	[12]
TiC conductive “clay”	-	-	900	-	115	[13]
Compressed Activated microwave Expanded Graphite Oxide	Ionic Liquid	147	110	227	173	[14]
Activated Carbon	Deep Eutectic	140	70 ^a^	260	130 ^a^	[15]
Vanadium Oxide and Polymer	Gel	412	-	295	-	[16]
Graphene	Ionic Liquid	-	-	306	153 ^a^	[17]
Carbon nanotubes on Carbon nanofibers	Ionic Liquid	-	-	356	178 ^a^	[18]
Graphene	Organic	298	212	457	324	[19]
Ceramic, Electrostatic Capacitors						
Dielectric Material	F·g^−1^	F·cm^−3^		J·g^−1^	J·cm^−3^	Reference
Aqueous solution of sodium nitrate in titania nanotubes	34	114		70	230	This work
Polymer	-	-		-	27	[20]
Lead lanthanum zirconate titanate	-	-		-	22	[21]
Barium strontium titanate nanowires	-	-		-	15	[22]
Barium titanate nanocubes	-	-		-	5	[23]

^a^ Volumetric values are estimated assuming a bulk density of 0.5 g·cm^3^ for carbonaceous electrodes [14]. Note: all values of energy density are either taken from the original paper or calculated using the highest operational voltage shown therein.

Exemplary of the above argument is the difference between the dielectric behavior of powder SDM materials and tube SDM materials. In the former it is postulated the super dielectric behavior arises from dipole formation in liquid drops, containing dissolved salts, in the pores of the powder. The addition of more material simply adds more aqueous-pore filling-ion containing drops. Thus, the number of “dipoles” in the dielectric is exactly proportional to the amount of dielectric. Also, all dipoles are equivalent, on an average basis, in terms of ion concentration and length. Thus, it is anticipated that the dielectric constant will be independent of the dielectric thickness, as discussed, and demonstrated, in an earlier publication [2]. In contrast, the model presented in this paper indicates that the dielectric constant for tube SDM will be a function of tube length because the character of each dipole changes with length. As the model notes (Section 2.6), both the length and the amount of salt in each “dipole” increase with tube length. Specifically, the dielectric constant should be proportional to tube length squared (Figure 3).

The argument developed above suggests a simple test: measure the “dielectric constant” value as a function of tube length. As shown in Figure 3 the value of the dielectric constant does increase in a fashion consistent with the model. In effect, the measurements made in this work indicate each TSDM of a different length is effectively a “unique” material; the longer the tubes, for a given salt concentration, the higher the dielectric constant. Conclusion: the model of dielectric value proportionality to tube length (Section 2.6) is consistent with all observations.

Another unique aspect of TSDM is the observed contrast with other aqueous dielectrics: the maximum operating voltage is not limited to the voltage at which ions are produced by the electrolytic decomposition of water. Finally, it needs to be emphasized that the oxide tubes are insulating the conducting electrodes (underlaying unanodized titanium on one side and graphite on the other) from one another, and the solution from one of the electrodes, eliminating the possibility of electron conduction and the occurrence of electrochemical reactions. Thus, this multi-material mixture is a dielectric material.

### 2.4. Primary Empirical Findings

The primary findings of the present work are the following: (i) TSDM had intrinsic dielectric constants >1.0 × 10^5^ in all cases, thus making this material a “super dielectric”; (ii) Direct energy measurement showed TSDM based capacitors formed with water containing saturated salt concentrations consistently stored/delivered >215 J·cm^−3^ of dielectric material. This is far higher than ever observed for electrostatic capacitors; (iii) In all cases the maximum “capacitive” voltage was approximately 2.2 volts; (iv) The energy density was nearly independent of tube length when all other parameters were fixed; (v) The energy density was not a simple function of salt concentration.

There is some discussion in the literature of the best approach to evaluation of novel capacitors [24]. Energy storage clearly cannot be extrapolated from a measure of capacitance determined at a specific voltage (generally zero volts) such as that delivered by impedance spectroscopy [1,2,25,26]. Indeed, in this work, and as reviewed elsewhere [1,2], it is clearly shown that dielectric constant varies with voltage. In some recent work an alternative approach was advocated. “Farads/volume”, rather than energy stored, was employed to be the mark of capacitor performance [13,27]. Clearly, in the present case this could be quite misleading. For example, the 10 micron sample filled with a saturated salt solution (Table 1) is rated as a 750 F·cm^−3^ capacitor below 300 mV, but is “only” a 150 F·cm^−3^ capacitor between 2 V and 300 mV.

We chose to emphasize energy storage as the metric of performance, and to use a classic method to determine energy storage: measure it directly over the full voltage operating range. In this fashion the ambiguity associated with extrapolations from measurement at a single voltage was eliminated. However, the method employed herein does not indicate how the capacitor might operate at higher frequency. Naturally, additional work is needed to fully characterize TSDM.

One finding demonstrates the value of the general approach taken to characterization of the capacitors in this study. To wit: voltage dependent dielectric values were consistently observed. A similar observation was made for powder based SDM at low frequency (*ca.* 1.0 × 10^−3^ Hz). Specifically, in both the present work and the earlier work there were regions of voltage of constant dielectric constant with “elbows” in the log curves (Equation (1), Figure 2) at which the dielectric constant changed sharply. Generally, the dielectric value below ~350 mV was between 3 and 5 times greater than that at higher voltage. Similarly, the dielectric constant increased by a factor of between 3 and 5 for the powder SDM for voltages below about 350 mV.

### 2.5. Energy Density

The energy densities of all four TSDM with saturated salt solutions were directly measured as exceeding 215 J·cm^−3^. This is of the same order as observed for many of the best electric double layer capacitors (EDLC), generally known as super or ultra capacitors (Table 3), and far better than the best commercial capacitors of the EDLC type (~30 J·cm^−3^). Also, relative to electrostatic capacitors, the class to which TSDM belong, the TDSM capacitors are far superior. This conclusion is not based on a single data point, but rather the reproducibility of the data, and the fact that each capacitor showed approximately the same energy density (±15% of the average) through at least four charge/discharge cycles. It is necessary to note that capacitors are generally selected for particular applications based on many properties, not just energy density. Thus the comparisons made here are not intended to be complete, but rather to suggest the value of further study of TSDM.

It must be noted that even in the class of electrostatic capacitors, a class of capacitors in which it appeared the underlying presumption was that barium titanate is the ultimate dielectric material [28,29], this report is not the only evidence of recent, dramatic improvement. For example barium titanate powders loaded with metal particles show higher dielectric values [30,31,32,33], and there are many reports of high dielectric values arising from “extrinisic” properties, particularly surface states, often called “colossal dielectric” materials [34,35,36,37,38,39]. Only recently was a much higher energy density reported for an electrostatic capacitor, and the material used was a polymer [20]. Clearly there is evidence that barium titanate is not the ultimate dielectric. The comparison to this kind of capacitor is natural because the material tested here is a type of dielectric material. The fact that the energy density of the TSDM is about one order of magnitude greater than that of all-solid dielectric capacitors does not mean TSDMs are “better” capacitors, provided that there are many different applications other than energy storage in which all-solid dielectric capacitors excel. A review of recent advances in energy density is given in Table 3.

### 2.6. Model

The dielectric behavior model for SDM developed in earlier papers applies to TSDM with some modifications. The key hypothesis of the model: the dielectric constant of a medium is proportional to the length and density of electric dipoles in the medium. In brief:

Dielectric constant ∝ Dipole Length × Dipole Charge × Dipole Density
(5)

In the earlier “powder” version of the model it was assumed that the dipole length is proportional to the average pore size in the powder medium, as the ionic separation/dipole length, should equal the pore diameter. The pore length, and hence the dipole length, does not change with the thickness of the dielectric layer. In contrast, in the TSDM version of the model the dipole lengths are proportional to the tube length. Thus, the dipole lengths are a linear function of the “thickness” of the dielectric. If true, for a given salt concentration (dipole density), the dielectric constants observed should be proportional to the length of the tubes.

Dielectric constant ∝ Tube Length × Dipole Charge × Dipole Density
(6)

Moreover, in the TSDM the dipole density will be proportional to the total number of free salt molecules. In turn, the number of free salt molecules will be proportional to the product of tube volume and the salt concentration. As the only parameter of the tubes that varies from sample to sample is the length, this leads to this version of the model:

Dielectric constant ∝ Tube Length × Dipole Charge·(Tube Length × Free Salt Concentration)
(7)

**Figure 4:**
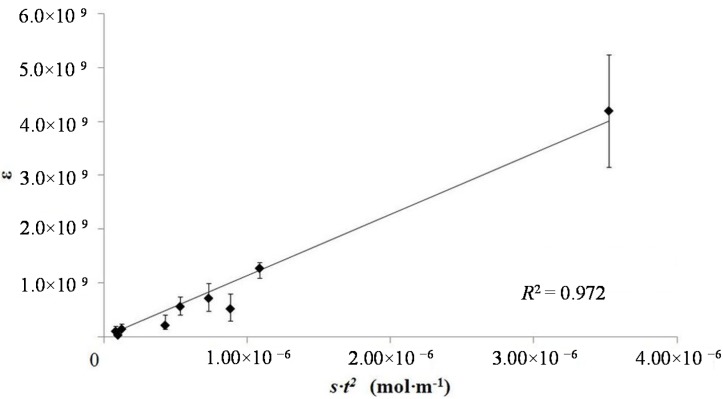
Model test plot. The plot indicates that the model developed in the text is consistent with the data. Further testing of the data suggests a different conclusion. That is the model of the impact of thickness on behavior is correct, but the salt dependence is not correct. The latter issue is not reflected in the above probably because the range of salt concentrations tested was not sufficiently large.

This is expressed as:

(8) $$\text{ε}\propto t^{2}\cdot S$$

where *t* is the tube length and *S* is the free salt concentration. In order to test this model, the dielectric constants measured from nine samples, with four different tube lengths and three salt concentrations, were plotted *vs.* *t*^2^·*S* (Figure 4). In this plot “*S*”, free salt, was assumed to be linear function of the aqueous phase salt concentration. There is a very good fit between model and observation as attested by an *R*^2^ value of a linear fit of ~0.97.

**Figure 5:**
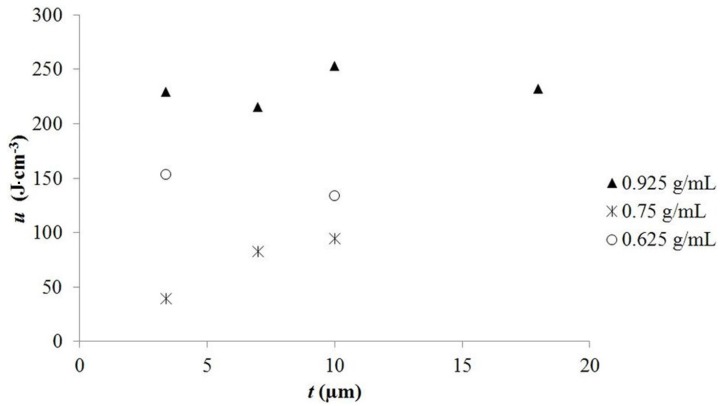
Energy density as a function of dielectric layer thickness. This figure shows that the energy density per dielectric volume in the “medium” voltage range is roughly independent of dielectric thickness, but is a non-linear function of salt concentration in the solution inside the tubes.

A plot of energy density as a function of tube length and salt concentration (Figure 4) reveals some observations consistent with the model, and others that are inconsistent with the model. In agreement with the model, the figure shows, all other parameters constant, that the energy density is nearly independent of tube length. For example, all tubes of a different length filled with saturated aqueous solutions of NaNO_3_ delivered >215 J·cm^−3^ of electrical energy. All data from nine capacitors show that for any given salt concentration, the tube length did not dramatically impact the energy density. However, the data show the model does not fit an energy density model (Equation (2)) in which free salt is a linear function of gross salt concentration. That assumption predicts a linear increase in both dielectric constant and energy density with increasing salt concentration. As shown in Figure 5 this is not the case. Capacitors made with the lowest salt concentration repeatedly showed higher energy density than those with a higher salt concentration. A similar result was reported in an earlier study of NaCl based superdielectrics [2]. The dielectric values, again counter to the model, were also not a linear function of salt concentration.

For the present, we offer a rough “plausibility” explanation for the observed non-linear “salt dependency”. Specifically, for reasons that are not clear, the number of ions participating in the formation of dipoles, that is, free salt ions, is not a linear function of salt concentration. At any given salt concentration there is a given fraction of ions free to form dipoles. This free fraction is not a linear function of salt concentration, but it is a consistent function of salt concentration. Support for the consistency argument is found in Figure 5: the energy density is nearly independent of tube length for any specific salt concentration. The only evidence in favor of “free salt model” is the consistency of the results for a given salt concentration. That is, the free salt ion model can explain why for a given salt concentration, the energy density at any tube length is virtually constant.

### 2.7. Breakdown Voltage

Another important parameter is the maximum “capacitive voltage”. Increasing this value is important because energy density is proportional to voltage squared (Equation (3)). Potentially improving this limit by determination and understanding of its source could dramatically improve stored energy density.

First, it is notable that a breakdown voltage of ~2.2 volts, at all tube lengths, is much higher than that found in EDLC employing aqueous electrolyte, <1.3 V. Standard electrochemistry indicates water will become highly conductive at this voltage. This suggests the voltage behavior does not reflect a breakdown due to current flow that occurs via the electrolysis of water at 1.3 volts. The observed high voltage also does not appear to reflect a breakdown voltage, that is, the field strength at which free charge carriers are produced. These inputs are used in the calculation of the breakdown voltage: (i) the breakdown voltage of distilled water is ~6.5 × 10^7^ V·m^−1^ [40]; (ii) salt solutions break down at a voltage about 10% lower than distilled water [41]; (iii) the water “length” over which breakdown occurs is the tube length. The third point alone makes water breakdown a dubious source for the limit to the capacitive voltage range. Indeed, if water breakdown were the source of the voltage limit breakdown, for the longest tubes would be 5 times greater than for the shortest tubes. In fact, the variation observed over all tube lengths was no greater than ten percent. Next, assuming as an upper bound that water containing sodium carbonate salt breaks down at the same voltage as distilled water we get a breakdown voltage for the 3.4 micron length tubes of 221 V. Assuming for a lower bound that the breakdown voltage for the salt water is only 10% of that of distilled water, we obtain a breakdown voltage of ~22 V. Clearly, the observed breakdown voltage is far lower than that expected for the salt water in the tubes.

Thus, a preliminary model is suggested, one consistent with the observed maximum voltage: a voltage-divider circuit exists. Some voltage drops across the oxide tubes between nanotubes and underlaying titanium, some across the salt water in the tubes. Regarding the former, it has been observed that titania nanotubes anodically grown in fluoride containing electrolytes form a Schottky junction with the metallic substate. The junction is a diode that prevents electron flow from the metal towards the tubes with a breakdown voltage around 2.5 V [42], above that observed herein. Thus, it is plausible that the observed maximum voltage (*ca*. 2.2 volts) corresponds to the case at which the voltage drop across the salt water section just equals the electrolysis voltage. However, the water breakdown would still produce a small current due to the requirement that hydrogen ions diffuse through a titania layer. This model is also supported by the fact that only when correctly polarized (Figure 3) to form a Schottky diode does the device acts as a capacitor. The improperly polarized capacitor stores no energy.

## 3. Experimental Section

### 3.1. Anodization Process

**Figure 6:**
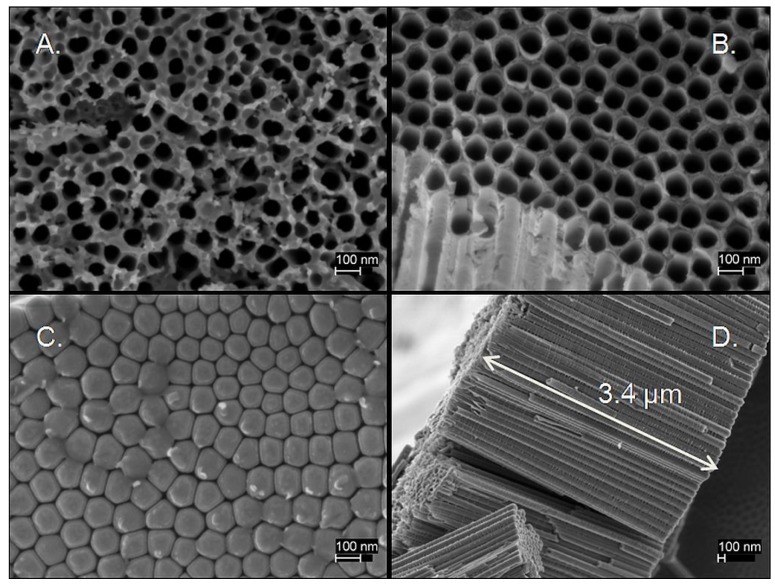
Scanning electron microscope (SEM) of TiO_2_ tubes. (**A**) Top view of nanotubes anodized for 60 min showing pores around 90 nm in diameter with surface debris; (**B**) Exposed intermediate cross section of nanotubes anodized for 260 min, showing the closely packed, ordered pores without surface debris. Notice that diameter is the same as in (**A**); (**C**) View of closed end, adjacent to parent foil; (**D**) Profile view of a group of the same TiO_2_ nanotubes where orientation and length is evident.

All dielectrics were generated by anodizing titanium foil, approx. 0.05 mm thick, using a process similar to one thoroughly described in the literature [43,44]. Specifically, anodization was carried out in a solution containing ammonium fluoride (0.25% *w*/*w*) and of water (2.75% *w*/*w*) in ethylene glycol using a titanium cathode (2 cm distant from the anode) and applying a constant DC voltage of 40 V for various time periods. The time was based on the desired tube length, and the simple observation that the titania tubes grow at a rate of approximately 1 μm/25 min in the particular conditions used. The longest tubes, 18 μm, were prepared using the same conditions except for the voltage and duration: 60 V for 1 h. Anodized anodes from this process were rinsed in ethanol and dried in air before and after the anodization. No thermal treatment was performed to the specimens. An example of the tubes formed by anodization is given in Figure 6. Clearly, the tubes formed from this process are very regular in structure and densely packed together. They are all oriented with the long axis perpendicular to the surface of the parent foil.

### 3.2. Assembly of Capacitors

First, in order to fill the tubes of the anodized material with an aqueous solution of sodium nitrate, the anodized foils were placed in a solution of sodium nitrate for 50 min at room temperature. Different levels of saturation of the solution were used in order to vary the salt concentration. In some cases full saturation, in others far less saturated solutions. The details for each capacitor are given in Table 1.

**Figure 7:**
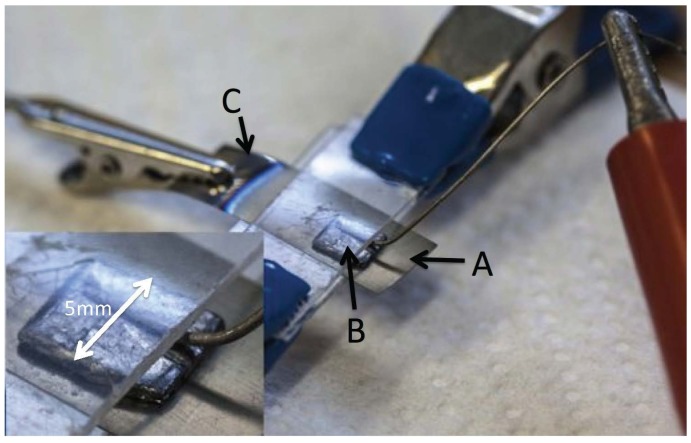
Assembled capacitor. (**A**) Anodized section of Ti foil; (**B**) Grafoil (5 mm × 6 mm) top electrode; (**C**) Metal section of Ti foil, bottom electrode. Inset shows polypropylene sheet compressing assembly, including tungsten wire, together. To maintain high humidity the entire assembly was kept in a plastic enclosure containing water saturated paper towels.

**Figure 8:**
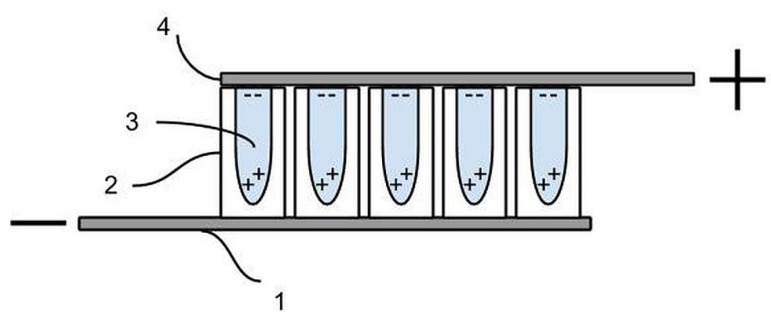
Capacitor, X-section. (**1**) Metal electrode, unanodized section of titanium metal foil; (**2**) Array of electrically insulating, oriented, micron/nano oxide tubes produced on titania foil via an anodization process; (**3**) Tubes filled with an aqueous solution with a high concentration of ions; (**4**) Open end of insulating tubes in contact with Grafoil, a second electrically conductive electrode. Schematically it is shown that within the ionic solution trapped in each tube the negative ions (**−**) will move toward the positive electrode, and the positive charged ions (**+**) will move toward the negative electrode.

At this point, two components of the capacitor were completed. One electrode is the metallic component of the anodized titanium foil. Indeed, part of the foil is not dipped into the anodizing solution, hence on this section no tubes form. The dielectric component consists of TiO_2_ nanotubes of various lengths, filled with an aqueous solution, at various levels of saturation, of sodium nitrate. The third and final component is a Grafoil electrode placed on top of a section of the anodized film. A rectangle of Grafoil (compressed natural graphite, 99.99% carbon [45,46]) measuring 5 × 6 mm^2^, or 6 × 6 mm^2^ on a side, and 0.3 mm thick was placed on top of the open end of the titania tubes. The metallic part of the anodized foil was connected to the negative terminal of a standard voltage supply, and the Grafoil sheet connected to the positive terminal of the voltage supply (see Figure 7 and Figure 8). This “sandwich” is the form for all capacitors tested in this study.

### 3.3. Determining Dielectric Constant and Energy Density

The focus on energy storage also determined the method employed to study the (effective) dielectric constant. Energy storage in ceramic capacitors is a field of considerable debate. Recently it was cogently argued that some capacitance data was improperly extrapolated without regard to saturation, maximum voltage, operating voltage and other factors [5], to yield dramatically exaggerated maximum energy densities for ferroelectric based capacitors. In order to avoid these difficulties the method employed herein allows a direct measure of the total energy output from the capacitor. Specifically, direct measurement of the RC time constant, over full charge and discharge cycles was selected. No algorithm is needed, just the time integration of the collected V^2^/R data. That is, like others we directly measured the RC time constant, over full charge and discharge cycles, to obtain the dielectric constant and the total energy [47].

Discharge data was analyzed to determine capacitance using the classic voltage decay equation for a capacitor discharging through a constant load (*R*) (Equation (1)).

### 3.4. Equivalent Circuit

**Figure 9:**
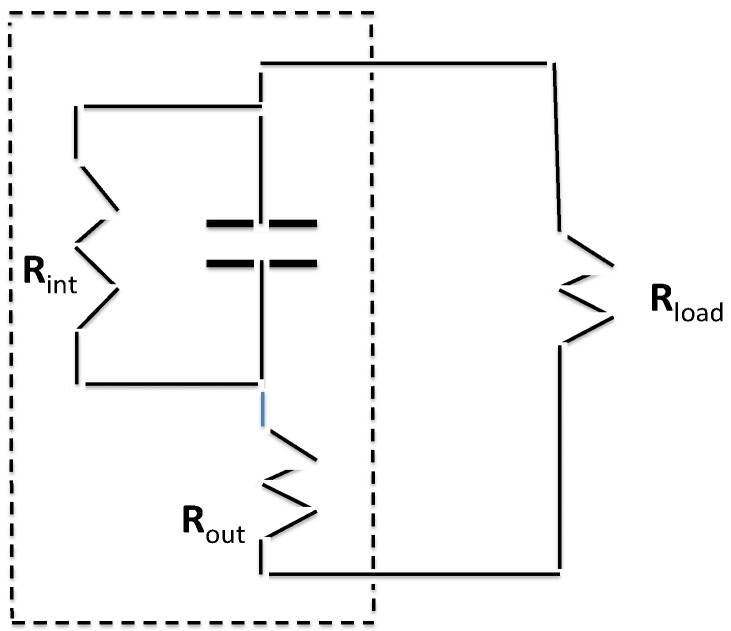
Assumed equivalent circuit. The area inside the dashed box represents the assumed equivalent circuit for low frequency behavior.

Two of the capacitors (labeled in Table 2) were tested to determine the approximate values of *R*_int_ and *R*_out_ in the presumed equivalent circuit shown in Figure 9. *R*_out_ was determined by removing *R*_load_ and replacing it with a 10 MΩ multi-meter in voltage mode. This caused the voltage to jump up a small amount. On the basis of repeated readings and the assumption of a voltage-divider circuit, an output resistance of 1 ± 0.1 kΩ was determined for both resistors at about 1.5 Volts charge. *R*_int_ was determined simply by removing *R*_load_ and episodically reading the remaining voltage over many hours. The value of *R*_int_ could be determined from an RC time constant computation. In both cases the value of *R*_int_ was determined to be 110 ± 10 kΩ. It is notable that the value of *R*_int_ is larger than many supercapacitors and *R*_out_ significantly smaller [48,49].

## 4. Conclusions

This study of the dielectric constants and electrical energy density of novel paradigm super (NPS) capacitors constructed with dielectric material consisting of porous anodic TiO_2_ filled with aqueous salt solutions supports the SDM hypothesis: porous insulating material in which the pores are filled with solutions of dissolved salts will have exceptional dielectric values, that is, superdielectrics with an intrinsic low frequency dielectric constant greater than 1.0 × 10^5^. Measurement showed that the dielectric constant of the materials studied herein, up to the discharge voltage of ~2.2 V, was greater than 1.0 × 10^7^ at ~0 Hz for all nine NPS capacitors studied, clearly making anodic TiO_2_ films filled with aqueous salt solutions superdielectrics.

The structure of the porous material studied is fundamentally different than that used in earlier SDM work, showing the generality of the SDM hypothesis. In particular, the anodization process employed produced a regular array of nanotubes about 90 nm across and of length equal to the thickness of the oxidized layer. That is, the anodized layer consisted of a regular array of pores perpendicular to the original metal surface and nearly equal in length to the distance between electrodes. In contrast, the powder material used in the earlier study had smaller pores that were an inherent property of the powder and clearly did not reach from electrode to electrode.

The energy density was remarkably high, consistent with the predictions of the SDM model. The data from nine NPS capacitors created with anodized titania films of different thicknesses and filled with aqueous solutions with a range of NaNO_3_ concentrations, showed energy density to be a complex function of salt concentration, with the highest energy density, >215 J·cm^−3^, corresponding to the use of a saturated salt solution. This value is far greater than the best traditional barium titanate-based capacitors (~10 J·cm^−3^), as well as the best new polymer-based dielectric capacitors (~25 J·cm^−3^). Clearly, this is better than the best commercial EDLC and on par with the best values reported in the scientific literature for novel EDLCs.
